# Characterization of the complete chloroplast genome of *Cornus bretschneideri* (cornaceae)

**DOI:** 10.1080/23802359.2019.1710281

**Published:** 2020-01-14

**Authors:** Xiaojuan Li, Qian Ma, Huakun Zhou, Yongsheng Yang, Huimei Li, Jiuli Wang

**Affiliations:** a Key Laboratory of Biotechnology and Analysis and Test in Qinghai-Tibet Plateau, Qinghai Nationalities University, Xining, China;; b Key Laboratory of Resource Chemistry and Eco-environmental Protection in Tibetan Plateau (Qinghai Nationalities University), State Ethnic Affairs Commission, Xining, China;; c Key Laboratory of Restoration Ecology in Cold Region of Qinghai Province, Northwest Institute of Plateau Biology, Chinese Academy of Sciences, Xining, China;; d Key Laboratory of Adaptation and Evolution of Plateau Biota, Northwest Institute of Plateau Biology, Chinese Academy of Sciences, Xining, China

**Keywords:** *Cornus bretschneideri*, chloroplast genome, Cornaceae, phylogenetic tree

## Abstract

*Cornus bretschneideri* L.Henry (Cornaceae), a shrub or small tree, is a potential horticultural plant or a soil-fixing plant. In this study, the complete sequence and characterization of the chloroplast genome of *C. bretschneideri* was studied. The size of the chloroplast genome is 158,270 bp in length, including a large single copy region (LSC) of 87,466 bp, a small single copy region (SSC) of 18,730 bp, and a pair of inverted repeat (IR) regions with 26,037 bp. The GC content of the chloroplast genome was 37.86%. Moreover, a total of 132 functional genes were annotated, including 87 protein-coding genes, 37 tRNA genes, and 8 rRNA genes. The neighbor-joining phylogenetic tree suggested that *C. bretschneideri* was closely related to *C. sanguinea* and *C. macrophylla*.

*Cornus bretschneideri* L. Henry (Cornales: Cornaceae), a shrub or small tree, is a potential horticultural plant or a soil-fixing plant. The genus *Cornus* consists of 58 species that are mainly distributed in the region of East Asia and North America, only two species are distributed in South America and one in tropical East Africa (Woźnicka et al. 2015). Recently, a series of chloroplast genome of *Cornus* and its allies were sequenced to conducted the phylogenomic analysis of Cornales (Fu et al. 2019). However, the phylogenetic location of *C. bretschneideri* has not been reported so far. Here, the complete chloroplast genome of *C. bretschneideri* (Genbank accession number: MN651479) was obtained based on high-throughput sequencing technology and the phylogenetic analysis of *C. bretschneideri* was carried out accordingly.

In this study, the samples of *C. bretschneideri* were collected from Tiebu Nature Reserve, Ruoergai County, Aba Prefecture, Sichuan Province, China (34.10°N, 103.10°E). The experiment and analysis scheme refers to Wang et al. (2019). Total DNA of *C. bretschneideri* was extracted from the dried, young leaves (about 0.3 g) with a modified CTAB method (Doyle and Doyle 1987). The voucher specimen (Specimen number: WangJL2019113) was kept in Herbarium of the Northwest Institute of Plateau Biology, Chinese Academy of Sciences (HNWP). Genome sequencing was performed using the Illumina HiSeq Platform (Illumina, San Diego, CA) at Genepioneer Biotechnologies Inc., Nanjing, China. Approximately 5.66 GB of clean data were yielded. The trimmed reads were mainly assembled by SPAdes (Bankevich et al. 2012). The assembled genome was annotated using CpGAVAS (Liu et al. 2012).

The complete chloroplast genome of *C. bretschneideri* is 158,270 bp in length with a typical quadripartite structure, containing a pair of inverted repeated (IR) regions of 26,037 bp, a large single copy (LSC) region of 87,466 bp, and a small single copy (SSC) region of 18,730 bp. The two IRs are separated by the LSC and the SSC. The GC content of the complete chloroplast genome was 37.86%. A total of 132 functional genes were annotated, including 8 rRNA genes, 37 tRNA genes, and 87 protein-coding genes. The rRNA genes, tRNA genes, and protein-coding genes account for 6.06%, 28.03%, and 65.90% of all annotated genes, respectively.

Phylogenetic relationships of *C. bretschneideri,* with 20 other species of *Cornus* and *Camptotheca acuminata* (Nyssaceae), were resolved by means of Neighbor-joining. Alignment was conducted using MAFFT (Katoh and Standley 2013; online version: https://mafft.cbrc.jp/alignment/server/). The Neighbor-joining tree was built using MEGA 7 (Kumar et al. 2016) with bootstrap set to 1000. The neighbor-joining phylogenetic tree suggested that *C. bretschneideri* was closely related to *C. sanguinea* and *C. macrophylla* (Figure 1).

**Figure 1.**
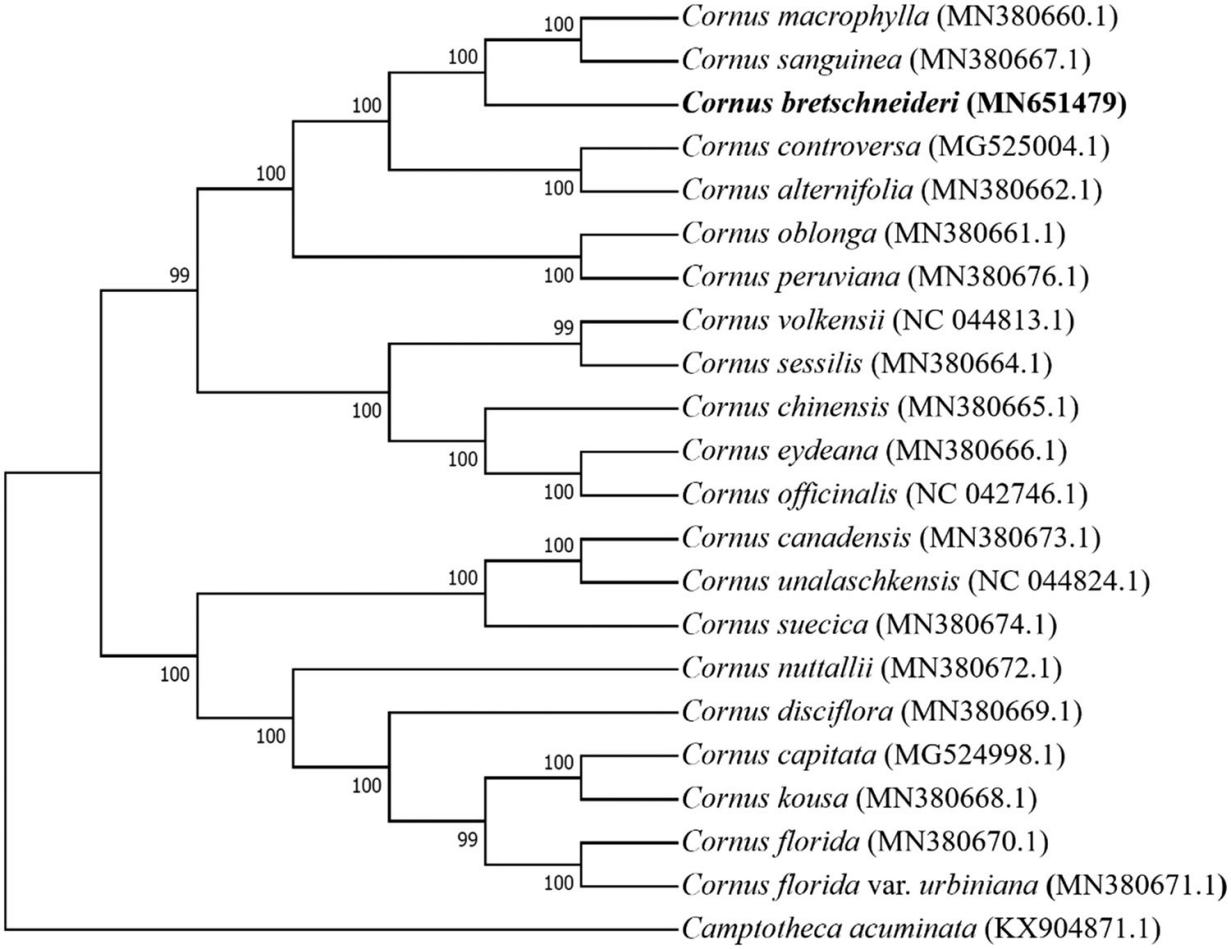
The Neighbor-joining phylogenetic tree based on 22 chloroplast genome sequences.
